# Supplementary material for: Graphene-Binding Peptide in Fusion with SARS-CoV-2 Antigen for Electrochemical Immunosensor Construction
Source: Biosensors (Basel). 2022 Oct 17;12(10):885. doi: 10.3390/bios12100885 (PMC9599560; doi:10.3390/bios12100885)
Supplement: Supplementary file 1 [file biosensors-12-00885-s001.zip › biosensors-1955813-supplementary.pdf]

Graphene-binding peptide in fusion with SARS-CoV-2 antigen for electrochemical immunosensor construction

Beatriz A. Braz[1,2], Manuel Hospinal-Santiani[1], Gustavo Martinz[2], Cristian S. Pinto[3], Aldo J. G. Zarbin[3], Breno C. B. Beirão[4], Vanete Thomaz-Soccol[1], Márcio F. Bergamini[2], Luiz H. Marcolino-Junior[2], Carlos R. Soccol[1].*

[1] Molecular Biology Laboratory, Graduate Program in Bioprocess Engineering and Biotechnology, Federal University of Paraná (UFPR), 81531-980, Curitiba, Paraná, Brazil.

[2] Laboratory of Electrochemical Sensors (LabSensE), Department of Chemistry, Federal University of Paraná (UFPR), CP 19032, 81531-980, Curitiba, Paraná, Brazil.

[3] Materials Chemistry Group (GQM), Department of Chemistry, Federal University of Paraná (UFPR). CP 19032, 81531-980 Curitiba, Paraná, Brazil.

[4] Graduate Program in Microbiology, Parasitology, and Pathology, Federal University of Paraná (UFPR), 81531-980, Curitiba, Paraná, Brazil.

*Corresponding author: luiz1berto@ufpr.br

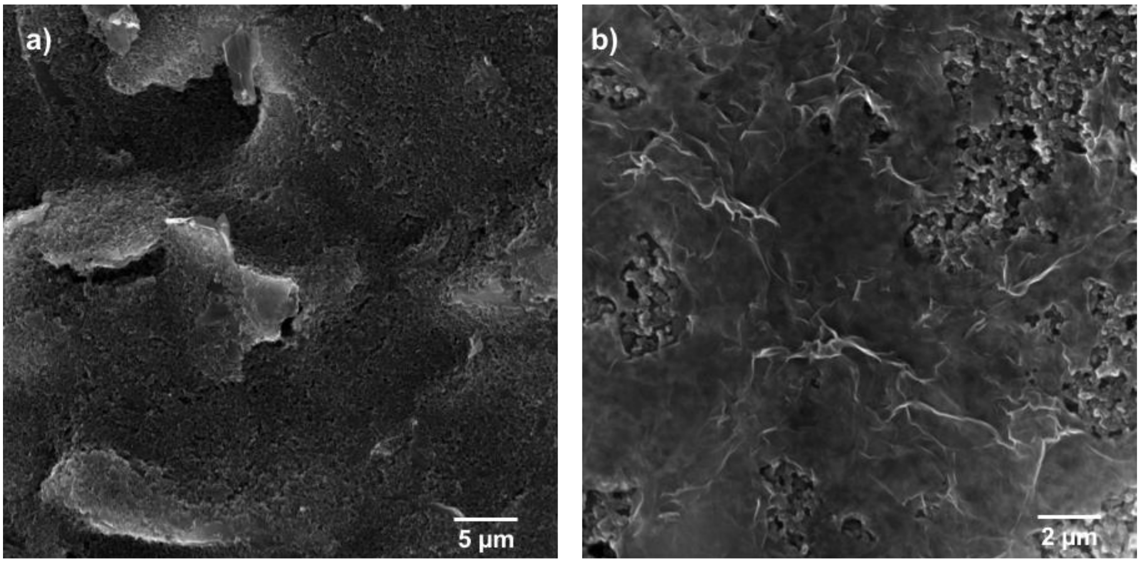

Figure S1. SEM images a) SPE – C surface and b) SPE with rGO.

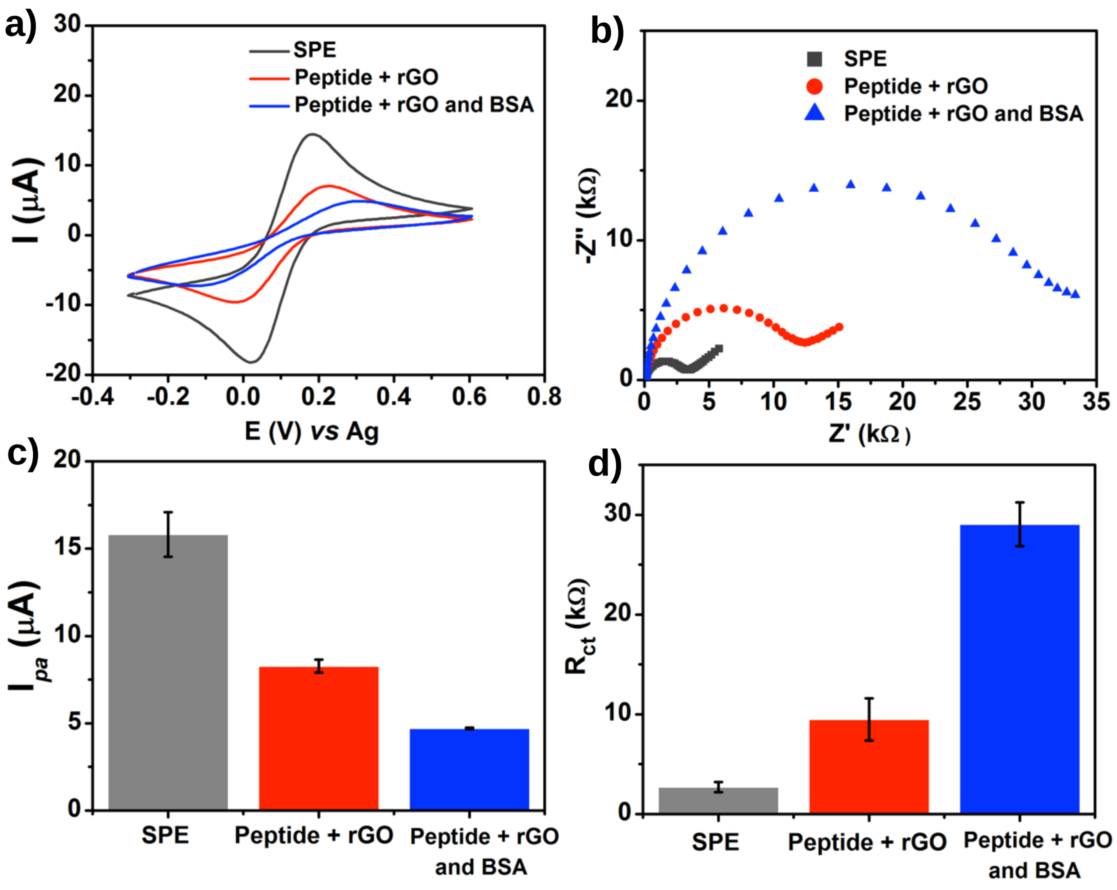

Figure S2. CVs and EIS measurements of each step of the immunosensor construction: a) CVs obtained for each step of the immunosensor construction with peptide, rGO and BSA, with 1 mmol L^{-1} $K_3[Fe(CN)_6]$ in PBS 0.1 mol L^{-1} at 50 mV s^{-1}. b) Nyquist plots obtained from EIS measurements in each step of the immunosensor building up, with 1 mmol L^{-1} $K_3[Fe(CN)_6]$ in PBS 0.1 mol L^{-1} and AC amplitude of 10 mV c) Summarized data obtained from probe anodic current peak (I_{pa}) to each step of building up the sensor (n=3, ±SD). d) Summarized data obtained from charge transfer resistance (R_{ct}) to each step of sensor construction (n=3, ±SD).

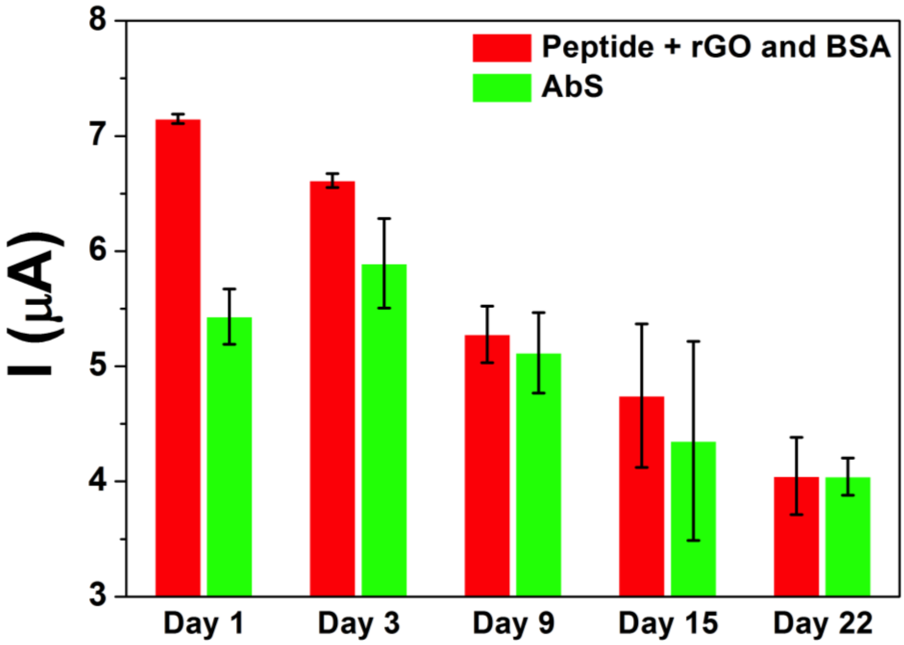

Figure S3. DPV data summarized for stability test of immunosensor readout stored over the time (n = 3, ± SD).
